# Dimethyl 1-(3-hy­droxy-2-iodo-1-phenyl­prop­yl)-1*H*-1,2,3-triazole-4,5-dicarboxyl­ate

**DOI:** 10.1107/S1600536812018193

**Published:** 2012-04-28

**Authors:** Hoong-Kun Fun, Tze Shyang Chia, Sivasubramanian Archana, Murugan Dinesh, Alagusundaram Ponnuswamy

**Affiliations:** a X-ray Crystallography Unit, School of Physics, Universiti Sains Malaysia, 11800 USM, Penang, Malaysia; b Department of Organic Chemistry, School of Chemistry, Madurai Kamaraj University, Madurai 625 021, Tamil Nadu, India

## Abstract

In the title compound, C_15_H_16_IN_3_O_5_, the central triazole ring is essentially planar (r.m.s deviation = 0.0034 Å) and makes a dihedral angle of 70.14 (5)° with the pendant benzene ring. The mean planes of the two meth­oxy­carbonyl groups make dihedral angles of 22.52 (7) and 40.93 (4)° with the triazole ring. In the crystal, inversion dimers linked by pairs of O—H⋯O hydrogen bonds generate *R*_2_^2^(18) loops. The dimers are linked by C—H⋯O and C—H⋯N inter­actions into sheets lying parallel to the *ac* plane.

## Related literature

For background to the industrial applications of 1,2,3-tri­azoles, see: Wamhoff (1984 ▶). For the stability of the temperature controller used in the data collection, see: Cosier & Glazer (1986 ▶).
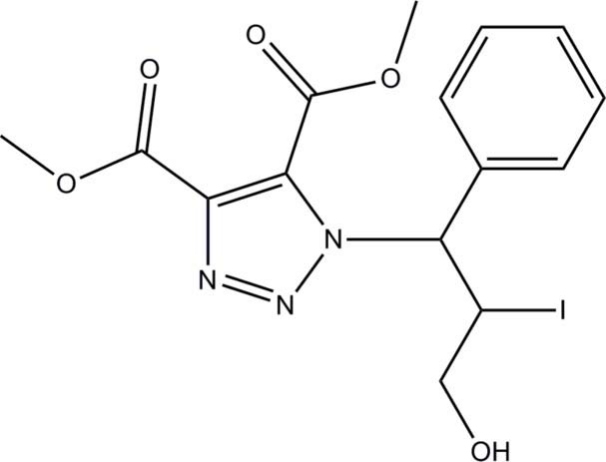


## Experimental

### 

#### Crystal data

C_15_H_16_IN_3_O_5_
*M* *_r_* = 445.21
Triclinic, 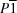

*a* = 8.0504 (3) Å
*b* = 9.6941 (4) Å
*c* = 11.1893 (4) Å
α = 106.426 (1)°
β = 91.798 (1)°
γ = 98.606 (1)°
*V* = 825.65 (5) Å^3^
*Z* = 2
Mo *K*α radiation
μ = 1.97 mm^−1^
*T* = 100 K
0.31 × 0.24 × 0.14 mm

#### Data collection

Bruker APEX DUO CCD diffractometer
Absorption correction: multi-scan (*SADABS*; Bruker, 2009 ▶) *T*_min_ = 0.579, *T*_max_ = 0.763
21676 measured reflections
5934 independent reflections
5827 reflections with *I* > 2σ(*I*)
*R*_int_ = 0.015

#### Refinement

*R*[*F* ^2^ > 2σ(*F* ^2^)] = 0.015
*wR*(*F* ^2^) = 0.038
*S* = 1.10
5934 reflections
223 parameters
H atoms treated by a mixture of independent and constrained refinement
Δρ_max_ = 0.49 e Å^−3^
Δρ_min_ = −0.92 e Å^−3^

### 

Data collection: *APEX2* (Bruker, 2009 ▶); cell refinement: *SAINT* (Bruker, 2009 ▶); data reduction: *SAINT*; program(s) used to solve structure: *SHELXTL* (Sheldrick, 2008 ▶); program(s) used to refine structure: *SHELXTL*; molecular graphics: *SHELXTL*; software used to prepare material for publication: *SHELXTL* and *PLATON* (Spek, 2009 ▶).

## Supplementary Material

Crystal structure: contains datablock(s) global, I. DOI: 10.1107/S1600536812018193/hb6752sup1.cif

Structure factors: contains datablock(s) I. DOI: 10.1107/S1600536812018193/hb6752Isup2.hkl

Supplementary material file. DOI: 10.1107/S1600536812018193/hb6752Isup3.cml

Additional supplementary materials:  crystallographic information; 3D view; checkCIF report

## Figures and Tables

**Table 1:** Hydrogen-bond geometry (Å, °)

*D*—H⋯*A*	*D*—H	H⋯*A*	*D*⋯*A*	*D*—H⋯*A*
O5—H1*O*5⋯O3^i^	0.74 (2)	2.22 (2)	2.9213 (11)	159 (2)
C9—H9*A*⋯O1^ii^	0.97	2.57	3.3595 (13)	139
C13—H13*B*⋯O3^iii^	0.96	2.48	3.4375 (14)	174
C13—H13*C*⋯O5^i^	0.96	2.58	3.4247 (14)	147
C15—H15*C*⋯N2^iv^	0.96	2.62	3.4903 (14)	151

Symmetry codes: (i) 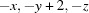; (ii) 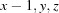; (iii) 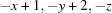; (iv) 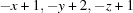.

**Fig. 1.**
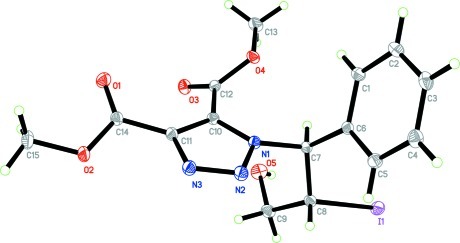
The molecular structure of the title compound with 50% probability displacement ellipsoids.

**Fig. 2.**
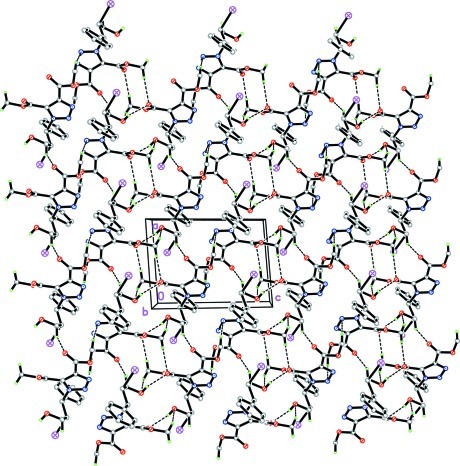
The crystal packing of the title compound. The dashed lines represent the hydrogen bonds. For clarity sake, hydrogen atoms not involved in hydrogen bonding have been omitted.

## supplementary crystallographic information

### Comment

1,2,3-Triazoles are important in a wide range of industrial applications such as agrochemicals, corrosion inhibitors, dyes, optical brighteners as well as biologically active agents (Wamhoff *et al.*, 1984). As part of our sudies in this area, we now describe the synthesis and structure of the title compound, (I) (Fig. 1).

The central triazole ring (N1–N3/C10/C11) is essentially planar [r.m.s deviation = 0.0034 Å] and makes a dihedral angle of 70.14 (5)° with the terminal benzene ring (C1–C6). The mean planes of the two methyl carboxylate groups (O1/O2/C14/C15 with maximum deviation = 0.0199 (6) Å at atom C14 and O3/O4/C12/C13 with maximum deviation = 0.0060 (6) Å at atom C12) make dihedral angles of 22.52 (7) and 40.93 (4)°, respectively with the triazole ring.

In the crystal (Fig. 2), the molecules are linked by O5—H1O5···O3, C9—H9A···O1, C13—H13B···O3, C13—H13C···O5 and C15—H15C···N2 hydrogen bonds (Table 1) into sheets parallel to *ac* plane.

### Experimental

A mixture of 3-azido-2-iodo-3-phenylpropan-1-ol (0.3 g, 0.99 mmol) and dimethyl but-2-ynedioate (0.14 g, 0.99 mmol) was heated to reflux in toluene for 3 h. The solvent was evaporated under reduced pressure to afford the crude reaction mass which was then subjected to column chromatography using silica gel (60–120 mesh) as the stationary phase and petroleum ether:ethyl acetate (80:20) as the mobile phase to give colourless blocks of (I) by slow recrystallization from the eluant. Yield: 0.35 g (81%); *M*.p.: 129–130 °C.

### Refinement

Atom H1O5 was located from difference fourier map and refined freely [O—H = 0.74 (2) Å]. The remaining H atoms were positioned geometrically [C—H = 0.93 0.96, 0.97 and 0.98 Å] and refined using a riding model with *U*_iso_(H) = 1.2 or 1.5 *U*_eq_(C). A rotating group model was applied to the methyl groups. Sixteen outliers (5 - 1 4), (-4 1 6), (-4 2 5), (-4 3 4), (-2 3 0), (-4 1 0), (-4 4 4), (-2 - 1 3), (2 1 2), (-2 2 2), (-1 4 0), (-4 - 2 5), (5 0 4), (3 - 1 3), (-3 1 1) and (2 - 4 3) were omitted.

### Crystal data

**Table:**

C_15_H_16_IN_3_O_5_	*Z* = 2
*M**_r_* = 445.21	*F*(000) = 440
Triclinic, *P*1	*D*_x_ = 1.791 Mg m^−^^3^
Hall symbol: -P 1	Mo *K*α radiation, λ = 0.71073 Å
*a* = 8.0504 (3) Å	Cell parameters from 9309 reflections
*b* = 9.6941 (4) Å	θ = 2.2–32.6°
*c* = 11.1893 (4) Å	µ = 1.97 mm^−^^1^
α = 106.426 (1)°	*T* = 100 K
β = 91.798 (1)°	Block, colourless
γ = 98.606 (1)°	0.31 × 0.24 × 0.14 mm
*V* = 825.65 (5) Å^3^	

### Data collection

**Table:**

Bruker APEX DUO CCD diffractometer	5934 independent reflections
Radiation source: fine-focus sealed tube	5827 reflections with *I* > 2σ(*I*)
Graphite monochromator	*R*_int_ = 0.015
φ and ω scans	θ_max_ = 32.6°, θ_min_ = 1.9°
Absorption correction: multi-scan (*SADABS*; Bruker, 2009)	*h* = −12→11
*T*_min_ = 0.579, *T*_max_ = 0.763	*k* = −14→14
21676 measured reflections	*l* = −16→16

### Refinement

**Table:**

Refinement on *F*^2^	Primary atom site location: structure-invariant direct methods
Least-squares matrix: full	Secondary atom site location: difference Fourier map
*R*[*F*^2^ > 2σ(*F*^2^)] = 0.015	Hydrogen site location: inferred from neighbouring sites
*wR*(*F*^2^) = 0.038	H atoms treated by a mixture of independent and constrained refinement
*S* = 1.10	*w* = 1/[σ^2^(*F*_o_^2^) + (0.0171*P*)^2^ + 0.3086*P*] where *P* = (*F*_o_^2^ + 2*F*_c_^2^)/3
5934 reflections	(Δ/σ)_max_ = 0.002
223 parameters	Δρ_max_ = 0.49 e Å^−^^3^
0 restraints	Δρ_min_ = −0.92 e Å^−^^3^

### Special details

**Table:**

Experimental. The crystal was placed in the cold stream of an Oxford Cryosystems Cobra open-flow nitrogen cryostat (Cosier & Glazer, 1986) operating at 100.0 (1) K.
Geometry. All e.s.d.'s (except the e.s.d. in the dihedral angle between two l.s. planes) are estimated using the full covariance matrix. The cell e.s.d.'s are taken into account individually in the estimation of e.s.d.'s in distances, angles and torsion angles; correlations between e.s.d.'s in cell parameters are only used when they are defined by crystal symmetry. An approximate (isotropic) treatment of cell e.s.d.'s is used for estimating e.s.d.'s involving l.s. planes.
Refinement. Refinement of *F*^2^ against ALL reflections. The weighted *R*-factor *wR* and goodness of fit *S* are based on *F*^2^, conventional *R*-factors *R* are based on *F*, with *F* set to zero for negative *F*^2^. The threshold expression of *F*^2^ > σ(*F*^2^) is used only for calculating *R*-factors(gt) *etc*. and is not relevant to the choice of reflections for refinement. *R*-factors based on *F*^2^ are statistically about twice as large as those based on *F*, and *R*- factors based on ALL data will be even larger.

### Fractional atomic coordinates and isotropic or equivalent isotropic displacement parameters (Å^2^)

**Table:**

	*x*	*y*	*z*	*U*_iso_*/*U*_eq_	
I1	−0.382668 (7)	0.637377 (7)	0.141512 (5)	0.01603 (2)	
O1	0.55901 (10)	1.17479 (8)	0.31744 (7)	0.01765 (13)	
O2	0.46348 (10)	1.25049 (8)	0.50897 (7)	0.01713 (13)	
O3	0.29674 (10)	1.04776 (7)	0.09582 (6)	0.01498 (12)	
O4	0.26687 (9)	0.80305 (7)	0.05704 (6)	0.01300 (12)	
O5	−0.09851 (11)	0.94656 (9)	0.12543 (7)	0.01876 (14)	
N1	0.13027 (10)	0.84350 (8)	0.29530 (7)	0.01084 (12)	
N2	0.13613 (11)	0.87602 (9)	0.42137 (7)	0.01341 (13)	
N3	0.25260 (11)	0.99050 (9)	0.46809 (7)	0.01367 (14)	
C1	0.11822 (13)	0.50289 (10)	0.15751 (9)	0.01454 (15)	
H1A	0.1649	0.5371	0.0942	0.017*	
C2	0.15365 (14)	0.37227 (10)	0.17255 (10)	0.01754 (17)	
H2A	0.2246	0.3202	0.1202	0.021*	
C3	0.08244 (14)	0.31994 (10)	0.26621 (10)	0.01701 (17)	
H3A	0.1065	0.2332	0.2771	0.020*	
C4	−0.02472 (14)	0.39767 (10)	0.34358 (9)	0.01666 (17)	
H4A	−0.0740	0.3616	0.4051	0.020*	
C5	−0.05871 (13)	0.52943 (10)	0.32951 (9)	0.01423 (15)	
H5A	−0.1295	0.5814	0.3822	0.017*	
C6	0.01325 (11)	0.58325 (9)	0.23655 (8)	0.01099 (14)	
C7	−0.00868 (11)	0.72960 (9)	0.22038 (8)	0.01059 (13)	
H7A	0.0028	0.7236	0.1322	0.013*	
C8	−0.17309 (12)	0.78729 (9)	0.25651 (8)	0.01215 (14)	
H8A	−0.1878	0.7938	0.3444	0.015*	
C9	−0.16977 (13)	0.93812 (10)	0.23842 (9)	0.01566 (16)	
H9A	−0.2837	0.9592	0.2369	0.019*	
H9B	−0.1044	1.0111	0.3085	0.019*	
C10	0.24390 (12)	0.94051 (9)	0.26041 (8)	0.01084 (14)	
C11	0.32129 (12)	1.03422 (9)	0.37215 (8)	0.01193 (14)	
C12	0.27066 (11)	0.93847 (9)	0.12878 (8)	0.01094 (14)	
C13	0.30152 (14)	0.78729 (11)	−0.07305 (8)	0.01628 (16)	
H13A	0.2970	0.6859	−0.1166	0.024*	
H13B	0.4116	0.8397	−0.0761	0.024*	
H13C	0.2187	0.8259	−0.1119	0.024*	
C14	0.46100 (12)	1.15881 (10)	0.39366 (8)	0.01275 (14)	
C15	0.60447 (14)	1.36969 (11)	0.54233 (10)	0.01921 (18)	
H15A	0.5865	1.4391	0.6191	0.029*	
H15B	0.6140	1.4164	0.4772	0.029*	
H15C	0.7063	1.3326	0.5527	0.029*	
H1O5	−0.166 (3)	0.934 (2)	0.0748 (18)	0.032 (5)*	

### Atomic displacement parameters (Å^2^)

**Table:**

	*U*^11^	*U*^22^	*U*^33^	*U*^12^	*U*^13^	*U*^23^
I1	0.01068 (3)	0.01859 (3)	0.01613 (3)	0.00087 (2)	−0.00104 (2)	0.00174 (2)
O1	0.0165 (3)	0.0192 (3)	0.0152 (3)	−0.0009 (3)	0.0032 (3)	0.0036 (2)
O2	0.0163 (3)	0.0158 (3)	0.0135 (3)	−0.0035 (3)	0.0017 (2)	−0.0018 (2)
O3	0.0186 (3)	0.0131 (3)	0.0134 (3)	0.0007 (2)	0.0005 (2)	0.0052 (2)
O4	0.0170 (3)	0.0114 (3)	0.0099 (3)	0.0024 (2)	0.0029 (2)	0.0018 (2)
O5	0.0203 (4)	0.0221 (3)	0.0174 (3)	0.0057 (3)	0.0010 (3)	0.0101 (3)
N1	0.0121 (3)	0.0110 (3)	0.0088 (3)	0.0010 (2)	0.0003 (2)	0.0024 (2)
N2	0.0157 (4)	0.0143 (3)	0.0089 (3)	0.0004 (3)	0.0004 (3)	0.0024 (2)
N3	0.0148 (4)	0.0140 (3)	0.0108 (3)	0.0000 (3)	0.0007 (3)	0.0024 (2)
C1	0.0162 (4)	0.0122 (3)	0.0152 (4)	0.0034 (3)	0.0042 (3)	0.0033 (3)
C2	0.0192 (4)	0.0130 (4)	0.0205 (4)	0.0052 (3)	0.0041 (3)	0.0033 (3)
C3	0.0191 (4)	0.0117 (3)	0.0202 (4)	0.0019 (3)	−0.0010 (3)	0.0051 (3)
C4	0.0205 (4)	0.0150 (4)	0.0151 (4)	0.0012 (3)	0.0009 (3)	0.0064 (3)
C5	0.0159 (4)	0.0141 (3)	0.0130 (3)	0.0023 (3)	0.0024 (3)	0.0044 (3)
C6	0.0111 (4)	0.0102 (3)	0.0107 (3)	0.0009 (3)	0.0001 (3)	0.0021 (3)
C7	0.0104 (3)	0.0103 (3)	0.0099 (3)	0.0009 (3)	0.0001 (3)	0.0017 (3)
C8	0.0117 (4)	0.0123 (3)	0.0114 (3)	0.0022 (3)	0.0005 (3)	0.0017 (3)
C9	0.0172 (4)	0.0133 (3)	0.0172 (4)	0.0052 (3)	0.0023 (3)	0.0041 (3)
C10	0.0118 (4)	0.0108 (3)	0.0098 (3)	0.0017 (3)	0.0007 (3)	0.0029 (3)
C11	0.0124 (4)	0.0117 (3)	0.0103 (3)	0.0009 (3)	0.0005 (3)	0.0017 (3)
C12	0.0102 (3)	0.0122 (3)	0.0095 (3)	0.0011 (3)	0.0003 (3)	0.0022 (3)
C13	0.0218 (5)	0.0167 (4)	0.0094 (3)	0.0030 (3)	0.0033 (3)	0.0020 (3)
C14	0.0126 (4)	0.0123 (3)	0.0120 (3)	0.0010 (3)	−0.0006 (3)	0.0020 (3)
C15	0.0171 (4)	0.0160 (4)	0.0197 (4)	−0.0040 (3)	−0.0024 (3)	0.0013 (3)

### Geometric parameters (Å, º)

**Table:**

I1—C8	2.1649 (9)	C4—C5	1.3953 (13)
O1—C14	1.2059 (11)	C4—H4A	0.9300
O2—C14	1.3414 (11)	C5—C6	1.3942 (12)
O2—C15	1.4491 (12)	C5—H5A	0.9300
O3—C12	1.2091 (11)	C6—C7	1.5148 (12)
O4—C12	1.3264 (10)	C7—C8	1.5347 (13)
O4—C13	1.4602 (11)	C7—H7A	0.9800
O5—C9	1.4227 (12)	C8—C9	1.5283 (13)
O5—H1O5	0.74 (2)	C8—H8A	0.9800
N1—N2	1.3534 (10)	C9—H9A	0.9700
N1—C10	1.3588 (11)	C9—H9B	0.9700
N1—C7	1.4904 (12)	C10—C11	1.3842 (12)
N2—N3	1.3091 (11)	C10—C12	1.4902 (12)
N3—C11	1.3646 (11)	C11—C14	1.4811 (13)
C1—C2	1.3923 (13)	C13—H13A	0.9600
C1—C6	1.3991 (12)	C13—H13B	0.9600
C1—H1A	0.9300	C13—H13C	0.9600
C2—C3	1.3913 (14)	C15—H15A	0.9600
C2—H2A	0.9300	C15—H15B	0.9600
C3—C4	1.3907 (14)	C15—H15C	0.9600
C3—H3A	0.9300		
			
C14—O2—C15	114.80 (8)	C7—C8—I1	109.17 (6)
C12—O4—C13	115.88 (7)	C9—C8—H8A	109.1
C9—O5—H1O5	110.5 (15)	C7—C8—H8A	109.1
N2—N1—C10	110.40 (7)	I1—C8—H8A	109.1
N2—N1—C7	118.32 (7)	O5—C9—C8	111.51 (7)
C10—N1—C7	130.48 (7)	O5—C9—H9A	109.3
N3—N2—N1	108.01 (7)	C8—C9—H9A	109.3
N2—N3—C11	108.72 (7)	O5—C9—H9B	109.3
C2—C1—C6	120.75 (8)	C8—C9—H9B	109.3
C2—C1—H1A	119.6	H9A—C9—H9B	108.0
C6—C1—H1A	119.6	N1—C10—C11	104.27 (7)
C3—C2—C1	119.75 (9)	N1—C10—C12	124.96 (8)
C3—C2—H2A	120.1	C11—C10—C12	130.77 (8)
C1—C2—H2A	120.1	N3—C11—C10	108.59 (8)
C4—C3—C2	119.87 (9)	N3—C11—C14	122.16 (8)
C4—C3—H3A	120.1	C10—C11—C14	129.19 (8)
C2—C3—H3A	120.1	O3—C12—O4	126.17 (8)
C3—C4—C5	120.40 (9)	O3—C12—C10	123.15 (8)
C3—C4—H4A	119.8	O4—C12—C10	110.64 (7)
C5—C4—H4A	119.8	O4—C13—H13A	109.5
C6—C5—C4	120.08 (9)	O4—C13—H13B	109.5
C6—C5—H5A	120.0	H13A—C13—H13B	109.5
C4—C5—H5A	120.0	O4—C13—H13C	109.5
C5—C6—C1	119.13 (8)	H13A—C13—H13C	109.5
C5—C6—C7	123.26 (8)	H13B—C13—H13C	109.5
C1—C6—C7	117.53 (8)	O1—C14—O2	124.91 (9)
N1—C7—C6	109.14 (7)	O1—C14—C11	124.26 (8)
N1—C7—C8	106.17 (7)	O2—C14—C11	110.83 (8)
C6—C7—C8	118.54 (7)	O2—C15—H15A	109.5
N1—C7—H7A	107.5	O2—C15—H15B	109.5
C6—C7—H7A	107.5	H15A—C15—H15B	109.5
C8—C7—H7A	107.5	O2—C15—H15C	109.5
C9—C8—C7	111.09 (7)	H15A—C15—H15C	109.5
C9—C8—I1	109.34 (6)	H15B—C15—H15C	109.5
			
C10—N1—N2—N3	−0.86 (10)	I1—C8—C9—O5	−78.79 (9)
C7—N1—N2—N3	−171.70 (8)	N2—N1—C10—C11	0.45 (10)
N1—N2—N3—C11	0.91 (10)	C7—N1—C10—C11	169.83 (9)
C6—C1—C2—C3	0.76 (16)	N2—N1—C10—C12	−179.45 (8)
C1—C2—C3—C4	0.53 (16)	C7—N1—C10—C12	−10.07 (15)
C2—C3—C4—C5	−1.28 (16)	N2—N3—C11—C10	−0.64 (11)
C3—C4—C5—C6	0.75 (15)	N2—N3—C11—C14	−178.05 (8)
C4—C5—C6—C1	0.53 (14)	N1—C10—C11—N3	0.11 (10)
C4—C5—C6—C7	−176.10 (9)	C12—C10—C11—N3	180.00 (9)
C2—C1—C6—C5	−1.29 (15)	N1—C10—C11—C14	177.28 (9)
C2—C1—C6—C7	175.54 (9)	C12—C10—C11—C14	−2.83 (17)
N2—N1—C7—C6	−66.54 (10)	C13—O4—C12—O3	1.45 (14)
C10—N1—C7—C6	124.78 (9)	C13—O4—C12—C10	−176.52 (8)
N2—N1—C7—C8	62.31 (9)	N1—C10—C12—O3	140.62 (10)
C10—N1—C7—C8	−106.37 (10)	C11—C10—C12—O3	−39.26 (16)
C5—C6—C7—N1	90.62 (10)	N1—C10—C12—O4	−41.34 (12)
C1—C6—C7—N1	−86.06 (10)	C11—C10—C12—O4	138.79 (10)
C5—C6—C7—C8	−31.01 (13)	C15—O2—C14—O1	−4.72 (14)
C1—C6—C7—C8	152.31 (8)	C15—O2—C14—C11	175.14 (8)
N1—C7—C8—C9	54.79 (9)	N3—C11—C14—O1	156.03 (10)
C6—C7—C8—C9	177.90 (8)	C10—C11—C14—O1	−20.80 (16)
N1—C7—C8—I1	175.44 (5)	N3—C11—C14—O2	−23.83 (13)
C6—C7—C8—I1	−61.45 (9)	C10—C11—C14—O2	159.34 (9)
C7—C8—C9—O5	41.77 (11)		

### Hydrogen-bond geometry (Å, º)

**Table:**

*D*—H···*A*	*D*—H	H···*A*	*D*···*A*	*D*—H···*A*
O5—H1*O*5···O3^i^	0.74 (2)	2.22 (2)	2.9213 (11)	159 (2)
C9—H9*A*···O1^ii^	0.97	2.57	3.3595 (13)	139
C13—H13*B*···O3^iii^	0.96	2.48	3.4375 (14)	174
C13—H13*C*···O5^i^	0.96	2.58	3.4247 (14)	147
C15—H15*C*···N2^iv^	0.96	2.62	3.4903 (14)	151

Symmetry codes: (i) −*x*, −*y*+2, −*z*; (ii) *x*−1, *y*, *z*; (iii) −*x*+1, −*y*+2, −*z*; (iv) −*x*+1, −*y*+2, −*z*+1.
